# Effects of Dietary Habits on Markers of Oxidative Stress in Subjects with Hashimoto’s Thyroiditis: Comparison Between the Mediterranean Diet and a Gluten-Free Diet

**DOI:** 10.3390/nu17020363

**Published:** 2025-01-20

**Authors:** Martina Laganà, Tommaso Piticchio, Angela Alibrandi, Rosario Le Moli, Francesco Pallotti, Alfredo Campennì, Salvatore Cannavò, Francesco Frasca, Rosaria Maddalena Ruggeri

**Affiliations:** 1 Endocrine Unit, Department of Human Pathology of Adulthood and Childhood DETEV, University of Messina, 98125 Messina, Italy; martinalagana5@gmail.com (M.L.); salvo.cannavo@unime.it (S.C.); 2 Department of Medicine and Surgery, University of Enna “Kore”, 94100 Enna, Italy; tommaso.piticchio@unikore.it (T.P.); rosario.lemoli@unikore.it (R.L.M.); francesco.pallotti@unikore.it (F.P.); 3 Unit of Statistical and Mathematical Sciences, Department of Economics, University of Messina, 98125 Messina, Italy; angela.alibrandi@unime.it; 4 Unit of Nuclear Medicine, Department of Biomedical and Dental Sciences and Morpho-Functional Imaging, University of Messina, 98125 Messina, Italy; acampenni@unime.it; 5 Endocrinology Section, Department of Clinical and Experimental Medicine, Garibaldi Nesima Hospital, University of Catania, 95124 Catania, Italy; f.frasca@unict.it

**Keywords:** Mediterranean diet, gluten free diet, autoimmune thyroid diseases, oxidative stress, anti-oxidants, thyroiditis

## Abstract

Background: The Mediterranean diet (MedD) exerts anti-inflammatory and anti-oxidant effects that are beneficial in autoimmune thyroid diseases (ATD). Recently, a gluten-free diet (GFD) has been proposed for non-celiac patients with Hashimoto’s thyroiditis (HT), but its usefulness is under debate. The present pilot study evaluates the effects of these two dietary regimes, with a focus on redox homeostasis, in HT. Patients and Methods: 45 euthyroid HT patients (30 F; median age 42 years) were randomly assigned to different dietary regimes: MedD (*n* = 15), GFD (*n* = 15) and free diet (FD, *n* = 15). Thyroid function tests, autoantibodies, and oxidative stress markers (Advanced glycation end products, AGEs; glutathione peroxidase (GPx), thioredoxin reductase (TRxR), and total plasma antioxidant activity (TEAA) were measured at baseline and after 12 weeks. Results: In the MedD group, significantly lower values of AGEs and higher values of GPX, TRX and TEAA with anti-oxidant action were detected (*p* < 0.05) at 12 weeks compared to baseline, and compared to the GFD and FD groups, in which the oxidative stress parameters did not change significantly (*p* > 0.05). No significant differences in serum levels of TSH, FT4, Ab-Tg, Ab-TPO compared to baseline were found in any group. Conclusions: This pilot study confirms the protective effect of the MedD against oxidative stress, while a GFD does not significantly influence markers of oxidative stress and/or thyroid autoimmunity/function parameters.

## 1. Introduction

Autoimmune thyroiditis, also called Hashimoto’s thyroiditis (HT), represents the prototype of an organ-specific autoimmune disease, and is the result of the loss of immunological tolerance to self-antigens and consequent activation of immune responses against thyroid tissue. The underlying pathogenetic mechanisms remain elusive and not fully understood yet. Genetic predisposition, epigenetic modifications and environmental factors are implicated in development and further progression of HT in susceptible individuals [1].

The incidence and prevalence of HT, as well as other autoimmune disease (ADs), has increased over the past decades, mainly in the North Western developed countries rather than in the East and South of the world [2,3,4,5]. Such a geo-epidemiological trend suggests that environmental factors are significant triggers of increased autoimmunity since considerable changes have occurred in more highly industrialized and wealthier societies [2,3,4,5,6,7].

In particular, interest has focused on the Western lifestyle, characterized by a sedentary lifestyle, psychological stress, and changes in dietary habits. The so-called Western-style diet (WD) is rich in calories, trans fatty acids, and proteins, high in salt and refined sugars, and low in fibers, and is characterized by the consumption of processed foods [6,7,8,9]. This dietary regime has been associated with an increased risk of several inflammatory and immune-mediated disorders, including autoimmune diseases (ADs), through various mechanisms: low-grade chronic inflammation (the so-called meta-inflammation), immune system dysregulation, enhanced oxidative stress and intestinal dysbiosis [10,11,12,13,14,15,16]. Moreover, this dietary regime is often associated with excess fat mass and obesity, further contributing to meta-inflammation [11,17,18].

As a consequence, several studies have investigated the possible association between nutrition and autoimmunity, and various dietary regimes have been proposed as valuable therapeutic strategies in preventing/counteracting inflammation and autoimmunity [19,20,21,22].

In contrast to the WD model, the Mediterranean diet (MedDiet), a plant-based diet inspired to the food patterns typical of the olive-growing areas of the Mediterranean basin, has been proposes as a model of healthy eating [21,23].

The main characteristics of MedDiet include: high intake of food from vegetable sources (vegetables, legumes, fresh fruits, nuts, whole grains) and olive oil as the principal source of fat; moderate consumption of red wine with meals; low to moderate intake of animal based products, mainly represented by fish (typically, the small oily fishes), dairy products, poultry, and eggs with a limited quantity of red meat and processed meat products [23]. Thanks to its high content in natural antioxidants, fibers and micronutrients nutritional components, the Mediterranean Diet positively influences immune system function, gut microbiota composition, and redox balance, exerting anti-oxidants, anti-inflammatory, and immunomodulatory effects. Evidence exists in favor of the protective effects of the MedDiet against diseases non communicable disease associated with chronic inflammation, like diabetes, obesity, cardiovascular diseases, cancer, and autoimmune disorders, including thyroiditis [21,22,23,24].

More recently, a gluten-free diet (GFD), recommended for gluten-dependent diseases (i.e., coeliac disease), has also been suggested as a treatment for other, non-gluten-dependent autoimmune disoders, with contrasting and inconclusive results [25,26,27,28,29,30]. A GFD eliminates all but very small amounts of gluten, a mixture of storage proteins, the prolamins, found in many cereals (spelt, wheat, rye, oats, and barley). Derived flours and foods (pasta, bread, …) containing gluten are eliminated from the diet. The supposed benefits of GFD range from supporting digestive processes, to maintaining microbiota balance, contrasting overweight, and preventing inflammation and autoimmunity. However, data from the literature does not fully support these claims, and further research is needed [25,26,27,28,29,30].

This pilot study was aimed at investigating the effects of different dietary habits on indices of thyroid function/autoimmunity, and their relationship with redox homeostasis, comparing a GFD and the MedDiet, whose anti-inflammatory anti-oxidant effects are well known also where thyroid autoimmunity is concerned [13,21].

## 2. Materials and Methods

### 2.1. Study Design and Patients

This was a prospective, single-blind, randomized controlled study performed at the University Hospital G. Martino of Messina, Italy, over a six-month period. The study participants were selected from newly diagnosed HT patients aged 18–65 years that consecutively referred to our outpatient clinics from June 2023 to December 2023. A total of 45 adult HT patients (30 females and 15 males; median age, 42 years) consented to participate and were enrolled in the study. Given the small sample size, a block randomization procedure was used to assign participants to the three arms of dietary intervention. To ensure an equal number of male and female participants in each group, two different randomization sequences were generated for female and male patients.

Inclusion criteria were: a diagnosis of HT in accordance to the currently accepted criteria (positive TPO-Ab and/or Tg-Ab, and/or decreased echogenicity of the thyroid parenchyma on thyroid ultrasonography) [31]; no diagnosis nor symptoms of coeliac disease (CD); Body Mass Index (BMI) within 18.5 and 29.5 kg/m^2^; stable eating habits in the previous six months; no history of dietary interventions, and pharmacological treatments including antioxidant agents and/or vitamin supplements, in the preceding six months.

All patients were euthyroid at time of recruitment, and none of them were taking L-thyroxine or any drugs potentially interfering with thyroid function or affecting redox balance at the time of recruiting and during the previous six months.

Exclusion criteria were obesity (body mass index [BMI] > 30 kg/m^2^); diabetes mellitus; kidney failure; neoplastic disease; existence of any comorbid autoimmune, infectious, or inflammatory disease; current or past smoking history; alcohol abuse.

**Figure 1:**
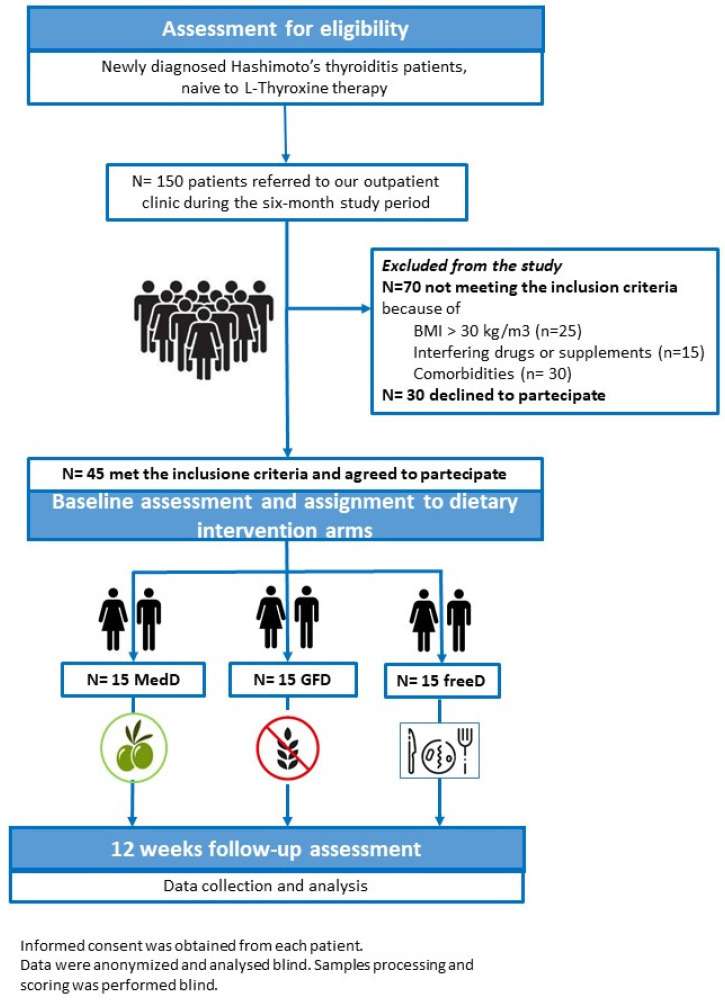
Flowchart of the study design.

All participants underwent thyroid hormone and antibody tests, and were given a questionnaire aimed at collecting data regarding lifestyle and dietary habits at baseline [13]. Also, all participants were asked if they used iodized salt to evaluate any difference in their iodine nutritional status. They agreed to provide a blood sample and to modify their dietary habits according to the study design. Then, each subject was randomly assigned to one of the three study “arms”, where each arm consisted of a different dietary intervention: GFD, MedD and free diet (freeD), that is no change in the subjects’ dietary habits (Figure 1). Patients received comprehensive information about the GFD and the correct MedDiet, and individualized nutrition advised by a registered dietitian, based on the current recommendations [32,33,34]. A non-consecutive three-day food record (2 days on weekdays and 1 day on weekends) was used at the end of the study, to evaluate adherence to the dietary intervention.

All subjects were informed of the study aims according to the Declaration of Helsinki and provided written informed consent.

### 2.2. Methods

Venous peripheral blood samples were collected into an anticoagulant solution containing EDTA after overnight fasting, centrifuged at 1450× *g* at 4 °C for 10 min, and stored in aliquots at −20 °C. Processing and scoring of samples were performed blind. At the end of the study, information regarding thyroid status and dietary interventions were linked to a code number and became available for statistical analysis.

All samples were processed centrally in the laboratory of our University Hospital of Messina. The main metabolic parameters (fasting glucose and insulin, lipids) were immediately measured. Serum TSH, free thyroxine (FT4), anti-thyroglobulin (Tg-Ab) and anti-thyroperoxidase (TPO-Ab) antibodies were measured by electrochemiluminescence immunoassay (ECLIA) using commercial kits for Elecsys 1010/2010 e modular analyticsE170 supplied by Roche Diagnostics (Roche Diagnostics, Mannheim, Germany). Normal values were as follows: TSH, 0.27–4.5 mIU/L; FT4, 9.0–22.0 pmol/L; Tg-Ab, 0–115 UI/L, TPO-Ab, 0–34 IU/mL. The intra- and/or the inter-assay CVs were <5 and <10%, respectively.

Advanced glycation end products (AGEs), markers of oxidative stress, were measured in serum samples as previously reported, and were expressed in arbitrary units (AU) per gram of protein (AU/g prot) [35]. Activity of antioxidant enzymes glutathione peroxidase (GPx), and thioredoxin reductase (TRxR), and total plasma antioxidant activity (TEAA) were measured in plasma samples as described elsewhere [13]. GPx and TRxR were measured in U/mL, whereas TEAA was expressed in mM TE, millimole of Trolox equivalents [13].

### 2.3. Statistical Analysis

The minimum sample size was calculated, with a confidence level of α = 0.050, assuming an expected difference of 0.25 between groups, and an expected residual standard deviation of about 0.5I. It was established that about 10 subjects per group would be needed to reach a power level of 0.80.

Statistical analyses were performed using SPSS (statistical package for social sciences) program for Window 22. A nonparametric approach was used since most numerical variables were not normally distributed, as verified by the Kolmogorov—Smirnov test. To assess the existence of significant differences among three groups, the Friedman test (for numerical parameters) and the chi-square, Fisher exact, or likelihood ratio tests were applied as appropriate (for categorical variables). In order to compare baseline vs. T1 timepoints, Wilcoxon test (for numerical parameters) and McNemar test (for dichotomous parameters) were applied. A *p*-value of <0.050 was considered to be statistically significant.

## 3. Results

Clinical, and biochemical features of our study population at recruitment are summarized in Table 1. Most of the study participants used iodized salt without differences between groups. All subjects were euthyroid (median TSH 1.8 mIU/ML; range 0.7–3.8; FT4 13.6 pm/L, range 12–16) at recruitment, and tested positive for TPO-Ab (median 245 IUI/L, range 45–4890) and/or Tg-Ab (402 IU/L, range 134–8070). When assigned to the three arms of dietary intervention, patients from each subgroup were sex and age-matched, and did not differ regarding the main anthropometric and metabolic parameters at baseline (T_0_) (Table 1). Similarly, there were no significant differences in TSH and FT4 values, nor in TPO-Ab and Tg-Ab levels, between groups at baseline. Also the parameters of oxidative stress under study were similar between groups at T_0_.

After 12 weeks of the assigned dietary regime (T_1_), blood parameters were re-evaluated in each subject to check for any significant changes in the three groups. No significant differences were found in serum levels of TSH, FT4, Tg-Ab and TPO-Ab at T_1_ compared to baseline (T_0_) in any group. In particular, concerning GFD, there were no significant changes in thyroid function tests at T_1_ compared to T_0_ (Table 2), although a decrease in TPO-Ab concentrations was observed, suggesting no relevant impact of gluten restriction on autoimmunity and thyroid function.

**Figure 2:**
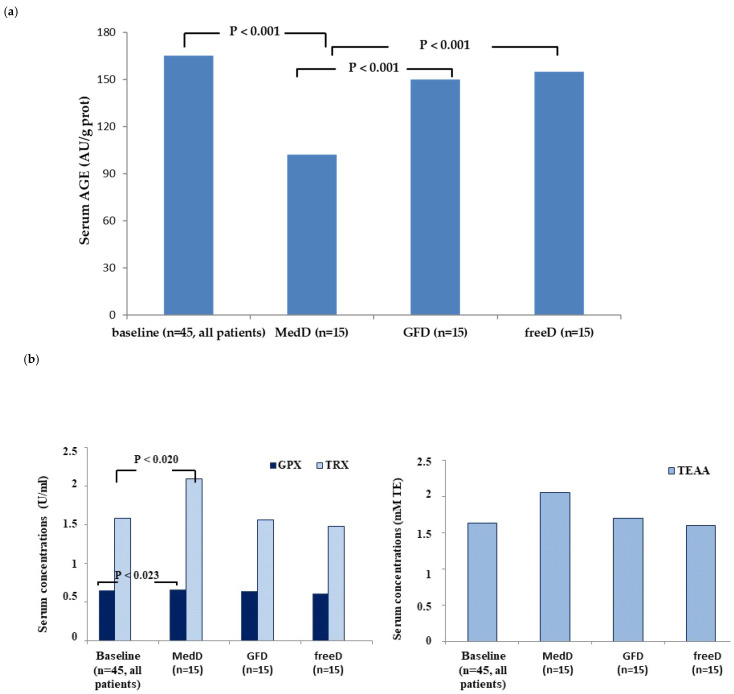
Serum levels of AGES (**a**) and antioxidants GPx, TRx and TEAA (**b**). Median values and range are reported in the Table 3.

Regarding oxidative stress parameters, we found significantly lower values of AGEs and higher values of the anti-oxidant GPX, TRX and TEAA in the MedD group (*p* < 0.001) at T_1_ compared to T_0_, and compared to the GFD and freeD groups at T_1_, in which the oxidative stress parameters did not change significantly compared to baseline (*p* > 0.05). (Table 3 and Figure 2).

## 4. Discussion

This pilot clinical study was aimed at assessing the actual effect of GFD and MedD on the oxidative stress of patients with HT. The main finding was the significant improvement in systemic redox balance after 12 weeks of MedD compared to baseline and GFD or freeD.

A shift toward a pro-oxidative state characterizes HT, even in euthyroid patients [35], and may contribute to the pathogenesis of thyroid autoimmunity. Indeed, the increased production of reactive oxygen radicals (ROS) due to environmental agents (iodine excess, radiation, drugs, pollutants, to mention a few) could induce a modification of tissue proteins, or might dysregulate the immune system, leading to loss of self-tolerance in genetically predisposed individuals [35]. Moreover, ROS accumulation promotes tissue inflammation and damage, thereby leading to increased rates of cell apoptosis, necrosis, and parenchymal destruction [30,35]. In this light, it is essential to identify approaches to restore the oxidative/antioxidative balance, beyond the well-known thyroid replacement therapy.

MedD is the dietary pattern that has shown the greatest benefits on chronic non-communicable diseases, including the autoimmune ones, and in several aspects of human health [29,36,37].

Our results agree with previous evidence suggesting a powerful anti-inflammatory and antioxidant action of MedDiet on the thyroid gland [21]. Previous observational studies reported that a high adherence to the MedD and a high intake of fruit, vegetables and cereals were protective against the risk of developing thyroid autoimmunity, while high consumption of red/processed meat and dairy products were significantly associated with an increased risk of thyroid autoimmunity and dysfunction [13,38,39,40]. In turn, it has been demonstrated that HT patients have higher serum AGE levels and reduced activity of the main antioxidant thyroid enzymes compared to controls, indicating a condition of oxidative stress [13]. The physiological mechanisms underlying the anti-inflammatory and anti-oxidant properties of MedDiet are not completely understood, but various food components are deemed to play a relevant role, including the phenolic compounds of wine (namely, resveratrol) exerting anti-oxidant effects; dietary ω-3 polyunsaturated fatty acids (PUFA), able to counteract inflammation through different mechanisms, and bioactive compounds of Extra Virgin Olive Oil (EVOO) [13]. Furthermore, most of the evidence in the literature supporting the benefits of dietary supplements for thyroid health stems from studies involving micronutrients abundant in the MedD, such as selenium, zinc, iron, vitamin D, and vitamin C [13,41,42,43,44,45]. Moreover, the high content in fiber of the MedDiet is useful to maintain a healthy gut microbiota [21,23]. All these food component have been demonstrated protective against the risk of developing thyroid autoimmunity

On the other hand, a GFD is recommended for CD, and a good compliance seems to also improve autoimmune thyroiditis and maintain euthyroid status in celiac patients with HT [46]. Despite this, its beneficial effects in patients with HT without a diagnosis or symptoms of CD remains controversial and widely debated.

A gluten-free dietary regimen has been proposed for HT treatment, even without CD, based on a series of epidemiological data and pathological hypothesis. (1) HT has shown a higher prevalence in celiac patients (10–30%) compared to the general population (5–10%) [47,48]; (2) HT was the most frequent disease described in patients with non-celiac gluten sensitivity [49]; (3) HT and CD share common genetic factors, in particular the susceptibility associated with HLA-DQ2 and HLA-DQ8 alleles [50,51]; (4) gluten could increase intestinal permeability, contributing to inflammation and the risk of autoimmunity in susceptible individuals [52]; and finally, (5) it has been hypothesized that GFD may exert a general systemic anti-inflammatory effect in extraintestinal autoimmune inflammatory diseases [53].

To date, available studies on this topic are limited and have been conducted on small cohorts. Therefore, a meta-analysis aimed to pool existing data was recently performed. The findings, based on a cohort of 87 patients who adhered to a gluten-free diet for nearly six months, indicated that significative changes in thyroid hormone and antibody levels were primarily observed in a subgroup of patients with a gluten-related condition (i.e., non-celiac gluten sensitivity or incidental finding of positive tissue transglutaminase antibodies without clinical symptoms or histological confirmation of CD) [54]. Moreover, a recent paper reported an improvement in FT3 and thyroid antibody levels after 12 weeks of a controlled diet for both GFD and MedD. Although each group consisted of only 10 people, the hormonal changes should be taken into account, considering the weight loss shown by the treated subjects, and the fact that the improvement of FT3 levels was significantly more important in the MedD group compared to the GFD group [55]. Finally, the most recent published paper on the subject reported that a GFD was able to exert an anti-inflammatory effect stated by the increase of anti-inflammatory mediators such as 17RS HDHA, 18RS HEPE, maresin 1, resolvin D1, and resolvin E1. In this study the GFD dietary regimen was supplemented with omega-3 eicosapentaenoic (EPA) and docosahexaenoic (DHA) acids alongside vegetables possessing anti-inflammatory properties. Hence, the authors attributed the increase of anti-inflammatory mediators to supplementation rather than gluten deprivation [26].

Our findings are consistent with these results, further supporting the strong antioxidant effects of the MedD on chronic inflammatory thyroid disease, and confirm the substantial ineffectiveness of a GFD in this clinical context.

In fact, the MedD is rich in poly-unsaturated fatty acids (PUFA) including omega-3 EPA and DHA, derived from mackerel, sardines, and anchovies, and omega-6 oleic and linoleic acids respectively derived from olives and walnuts. PUFA exert a systemic anti-inflammatory action also involving thyroid parenchyma [56]. Moreover, the MedD is also characterized by the consumption of whole cereals that are rich in phenols, poliphenols, phenolic acids, and minerals which play a key role in neutralizing free radicals. In particular, whole wheat contains phenols, vitamin E, and selenium [57]; spelt is rich in polyphenols, carotenoids, zinc, magnesium, and fiber [58]; and barley is a source of beta-glucans and phenolic compounds [59]. Furthermore, the fiber provided by these whole cereals is crucial for regulating the intestinal microbiota and preventing dysbiosis that could induce the molecular mimicry that triggers HT [60].

People who adhere to a GFD eliminate, either directly or indirectly, several sources of natural antioxidants and fibers. They often replace whole grains with white rice or refined gluten-free flours [61] and frequently avoid consuming oily nuts due to concern about gluten contamination. This fear induces them to restrict their diet to a limited variety of foods or to favor packaged gluten-free products, which increase the risk of an unhealthy diet, characterized by nutritional deficiencies and/or high levels of trans fats, salt, and a glycemic index [61,62]. Overall, there is currently not enough evidence to recommend a GFD to non-celiac patients with ATDs.

This study opens new scenarios about a comprehensive treatment of HT. Clinicians and patients need to be more aware about the crucial role of nutrition, in particular of the MedD, in the prevention and progression of chronic diseases. Finally, these findings could be further implemented with the use of clinical tools to assess inflammation [63].

The main limitations of this study were the small number of patients enrolled for each group and the short period of observation. Consequently, the study findings cannot be generalized to the broader population of patients with autoimmune thyroid disorders, and confirmation in a large samples series and other populations is required. However, the strict criteria for inclusion and the methods used, allowing for a direct investigation into systemic oxidant and antioxidant activity, must also be considered.

## 5. Conclusions

To the best of our knowledge this is the first study that directly compares the systemic oxidant and antioxidant activity between patients treated with two different dietary patterns. The MedD has shown a powerful antioxidant effect compared to baseline and a GFD. A GFD was ineffective in improving redox balance in patients with HT without CD. These preliminary findings should be confirmed on large series of HT patients and other populations, and should be enriched by the evaluation of additional clinical outcomes, such as quality of life and/or symptom relief and/or microbiota composition.

## Figures and Tables

**Table 1:** Demographic, anthropometric and biochemical features of our patients at baseline.

	MedD	GFD	freeD	*p* Value
Sex Male Female	5 10	5 10	5 10	
Age, yr median (range)	42 (25–65)	42 (24–65)	41 (22–65)	0.988
Body weight (kg)	63 (53–85)	63 (47–80)	62 (51–78)	0.979
BMI (kg/m^2^)	24 (18–29.5)	23 (20–29)	23 (21–29.5)	0.811
W/H Ratio	0.84 (0.70–0.95)	0.85 (0.7–0.94)	0.85 (0.75–0.90)	0.958
Fasting glucose (mg/dL)	78 (65–90)	80 (64–97)	79 (70–94)	0.924
Basal fasting insulin (µIU/L)	6.8 (4–10)	7.7 (4–13)	7.61 (5–11.5)	0.758
HOMA index	1.8 (1.3–2.9)	2.1 (1–3.4)	2 (1–3.5)	0.751
Total cholesterol (mg/dL)	171 (140–215)	178 (140–220)	179 (151–220)	0.816
LDL cholesterol (mg/dL)	89 (60–123)	91 (55–128)	90 (84–120)	0.983
HDL cholesterol (mg/dL)	62 (41–84)	59 (54–91)	59 (53–83)	0.877
Triglycerides (mg/dL)	86 (52–104)	89 (50–105)	87 (36–148)	0.977
TSH (mIU/L) *	1.6 (0.8–3.2)	1.8 (0.7–3.1)	1.85 (0.8–3.8)	0.858
FT4 (pml/L) *	13.6 (12–16)	13.8 (12.2–15.8)	13.2 (12–14.8)	0.636
Tg-Ab (IU/L)	469 (136–4000)	368 (150–8070)	402 (134–2659)	0.994
TPO-Ab (IU/L) *	244 (45–4890)	288 (140–3890)	255 (134–4126)	0.998

Data are expressed as median and range, in parenthesis. No statistically significant differences emerged between the three groups for any of the above reported parameters (*p* > 0.05, always) BMI, body mass index; WHR, waist hip ratio; HOMA, homeostatic model assessment index for estimation of insulin resistance. TSH, thyrotropin; FT4: free thyroixine; TPO-Ab, anti-thyroperoxidase antibodies; AGEs, advanced glycation end products; GR, glutathione reductase; GPx, glutathione peroxidase; SOD, superoxide dismutase; TEAA, total plasma antioxidant activity; TRxR, thioredoxin reductase. * Normal values are specified under Section 2.

**Table 2:** Thyroid function tests and autoantibodies at baseline and after 12 weeks of gluten-free diet *.

Parameters	Baseline (T0)	After 12 Weeks (T1)	*p* Value
TSH	1.8 (0.7–3.1)	1.8 (0.9–3.2)	0.929
FT4	13.6 (12–16)	13.5 (12–16.3)	0.496
Tg-Ab	368 (150–8070)	355 (133–3680)	0.207
TPO-Ab	288 (140–3890)	251.85 (110–3460)	0.020

* No differences between T_0_ and T_1_ were observed for routine blood parameters.

**Table 3:** Oxidative stress parameters at baseline and after 12 weeks of gluten-free diet *.

	OXIDATIVE STRESS PARAMETERS				
	AGE	GPx	TRx	TEAA	*p* Value ^#^
T_0_ all patients (*n* = 45)	165 (78–393)	0.59 (0.3–0.84)	1.58 (0.5–3.6)	1.63 (0–1.8)	NS
T_1_ MedD (*n* = 15)	102.7 (30–325)	0.68 (0.5–1.0)	2.09 (0.7–5.6)	2.06 (0.7–2.4)	vs. T_0_, <0.05 *
T_1_ GFD (*n* = 15)	165 (95–231)	0.63 (0.30–0.9)	1.56 (0.5–3.6)	1.7 (0.1–1.9)	vs. T_0_, NS
T_1_ freeD (*n* = 15)	168 (78–354)	0.60 (0.3–0.8)	1.48 (0.6–3.3)	1.63 (0–1.8)	vs. T_0_, NS

* Data are expressed as median and range in brackets. NS: not significant. **^#^** *p*-values T_0_ vs. T_1_: AGE, <0.001; GPx, 0.020; TRx, 0.023; TEAA, 0.002.

## Data Availability

The original contributions presented in this study are included in the article. Further inquiries can be directed to the corresponding author.
